# Synergistic Effect of Precursor and Interface Engineering Enables High Efficiencies in FAPbI_3_ Perovskite Solar Cells

**DOI:** 10.3390/ma16155352

**Published:** 2023-07-30

**Authors:** Sylvester Sahayaraj, Zbigniew Starowicz, Marcin Ziółek, Robert Socha, Łukasz Major, Anna Góral, Katarzyna Gawlińska-Nęcek, Marcin Palewicz, Andrzej Sikora, Tomasz Piasecki, Teodor Gotszalk, Marek Lipiński

**Affiliations:** 1 Institute of Metallurgy and Materials Science, Polish Academy of Sciences, 25 Reymonta St., 30-059 Krakow, Poland; sylvester.sahayaraj@cbrtp.pl (S.S.); starowicz.z@imim.pl (Z.S.); l.major@imim.pl (Ł.M.); goral.a@imim.pl (A.G.); gawlinska.k@imim.pl (K.G.-N.); 2 CBRTP SA Research and Development Center of Technology for Industry, Ludwika Waryńskiego 3A, 00-645 Warszawa, Poland; robert.socha@cbrtp.pl; 3 Faculty of Physics, Adam Mickiewicz University, 2 Uniwersytetu Poznańskiego St., 61-614 Poznan, Poland; marziol@amu.edu.pl; 4 Department of Nanometrology at the Faculty of Electronics, Photonics and Microsystems, Wrocław University of Science and Technology, 11/17 Janiszewskiego St., 50-372 Wroclaw, Poland; marcin.palewicz@pwr.edu.pl (M.P.); andrzej.sikora@pwr.edu.pl (A.S.); tomasz.piasecki@pwr.edu.pl (T.P.); teodor.gotszalk@pwr.edu.pl (T.G.)

**Keywords:** interface engineering, OAI, 2D perovskites, FAPbI_3_, perovskite solar cell

## Abstract

Formamidinium lead iodide (FAPbI_3_)-based perovskite solar cells have gained immense popularity over the last few years within the perovskite research community due to their incredible opto-electronic properties and the record power conversion efficiencies (PCEs) achieved by the solar cells. However, FAPbI_3_ is vulnerable to phase transitions even at room temperature, which cause structural instability and eventual device failure during operation. We performed post-treatment of the FAPbI_3_ surface with octyl ammonium iodide (OAI) in order to stabilize the active phase and preserve the crystal structure of FAPbI_3_. The formation of a 2D perovskite at the interface depends on the stoichiometry of the precursor. By optimizing the precursor stoichiometry and the concentration of OAI, we observe a synergistic effect, which results in improved power conversion efficiencies, reaching the best values of 22% on a glass substrate. Using physical and detailed optical analysis, we verify the presence of the 2D layer on the top of the 3D surface of the perovskite film.

## 1. Introduction

Hybrid organic–inorganic perovskite solar cells (PSCs) represented by the chemical formula ABX_3_ have been an interesting topic of research in the field of photovoltaics (PV) ever since they were first reported in 2009 with a PCE of 3.8% [1]. In the formula, A represents organic or inorganic cations such as methylammonium (MA^+^), cesium (Cs^+^), and formamidinium (FA^+^), B represents metals such as Pb, Sn, or Ge, and X represents a halide ion (I^−^, Cl^−^, or Br^−^). With the ease of fabrication, which involves simple processing steps such as spin coating and low-temperature annealing, coupled with the excellent opto-electronic properties of perovskite PV absorbers, rapid advances were made in the upcoming years, and at the moment, the maximum PCE for PSCs has reached an astonishing 26.1% (https://www.nrel.gov/pv/cell-efficiency.html, accessed on 25 July 2023) within a time span of approximately 13 years. For a major part of this time, most of the best PCEs reported in laboratories involved the use of MA^+^-based structures. Around the year 2020, the PCEs reported for MA^+^-based solar cells started to plateau, with PCEs not exceeding 22%, and at the same time, the frailties of MA^+^-based solar cells, such as poor thermal stability, were exposed [2]. Around this time, FA^+^-based PSCs of the FAPbI_3_ type started gaining prominence due to their superior thermal stability [3]. The attractive optical properties of FAPbI_3_, such as optimal bandgap (1.48 eV), high absorption coefficient, and long carrier lifetimes [4,5,6], make them a frontrunner in the perovskite research community. 

In spite of such strong merits, there are some grey areas associated with the material. FAPbI_3_ has been shown to have low structural stability [7,8,9]. The most suitable photoactive phase called the α phase (or the black phase) can undergo reversible phase transitions and can be converted to its polymorphs, namely the β and γ phases, at low temperatures [10]. At room temperature, the α phase tends to revert to the non-photoactive δ phase (the yellow phase), which has a wider band gap and very low absorption coefficient. The transition can be triggered due to the adsorption of O_2_ and H_2_O molecules, which causes distortion of the (PbI_6_)^4−^ octahedra and the eventual conversion from α to δ phase [11]. Over the years, a great deal of research has been undertaken to improve the structural stability of FAPbI_3_. Tuning the composition of the perovskite, i.e., the addition or partial replacement of the A site cation with other cations such as Cs^+^ [12], Rb^+^ [13], or partial replacement of I by Br [14], was expected to reduce the lattice distortion and stabilize the α phase. Although such efforts showed mild improvement in the PCE at the time, the resulting devices suffered from poor thermal and operational stability, primarily due to the formation of the δ phase, thereby suggesting that A site or halide doping could be ineffective for this purpose. Another promising area of work focused on the stabilization of FAPbI_3_ includes the use of additives to the precursor to moderate the crystallization of FAPbI_3_ and prevent the formation of the δ phase. The usage of chlorides in the perovskite precursor has been touted as an effective method to improve the grain size and morphology [15,16,17] of the perovskite films. Based on the success of previous reports, several Cl-containing compounds, such as methylammonium chloride (MACl) [18], formamidinium chloride (FACl) [19], and methylene diammonium chloride (MDACl_2_) [20,21], were used as additives to the perovskite precursor. In all cases, the film morphology of the perovskite was considerably improved. Both the PCE and the stability of the solar cells containing the additives showed greater improvement than the control samples. However, it is to be remembered that a bare perovskite film is still sensitive to the formation of defects due to interaction with the atmosphere, and a defective surface generally reduces the PCE of a solar cell due to non-radiative recombination that reduces the open circuit voltage (V_oc_). Depositing a thin film of a 2D perovskite made using long-chain alkyl/aryl ammonium organic cations on the surface can actually serve as a passivation layer, reducing interface recombination [22]. Additionally, the use of compounds with a long alkyl/aryl chain can stabilize the metastable perovskite phase in pure FAPbI_3_ without the need for additional cations or anions in the composition [22]. The presence of a long alkyl compound on the surface of the 3D film has been shown to promote hydrogen bonding with the (PbI_6_)^4−^octahedra, which enables the formation of self-assembled and vertically aligned (PbI_6_)^4−^octahedral slabs forming a two-dimensional perovskite [22]. Such a structure is resistant to atmospheric air and moisture due to the hydrophobicity of the large cation. The presence of a 3D/2D perovskite heterojunction stabilizes the α phase and prevents undesirable phase transitions. Therefore, engineering the interface of 3D-FAPbI_3_ with large organic cations is a promising area of work to improve the long-term structural stability of FAPbI_3_ PSCs. J. Suo et al. performed interfacial engineering of FAPbI_3_ perovskite films by forming a 2D perovskite with cyclohexyl methyl ammonium iodide (CMAI) [23]. N-hexyl trimethyl ammonium bromide (HTAB) was utilized by Jung et al. [24]. They carried out an in situ reaction, forming a thin layer of 2D perovskite on the surface of a low-band-gap perovskite absorber. In this work, we present the fabrication of FAPbI_3_ perovskites by conventional spin coating followed by interface engineering using octyl ammonium iodide (OAI). We discuss the conditions that lead to the formation of 2D perovskite at the interface and what happens when those conditions are not met. We demonstrate how precursor engineering and interface engineering work hand in hand, resulting in improved efficiencies of solar cells. Using detailed optical analysis, we illustrate the presence of a 2D perovskite layer in the structure and charge carrier kinetics in a 3D/2D graded heterojunction.

## 2. Materials and Methods

### 2.1. Materials

Lead iodide beads were purchased from Alfa Aesar. Formamidinium iodide and methylammonium chloride were purchased from Greatcell Solar. N2,N2,N2′,N2′,N7,N7,N7′,N7′-octakis(4-methoxyphenyl)-9,9′-spirobi [9H-fluorene]2,2′,7,7′-tetramine (Spiro-OMeTAD) was purchased from Sigma Aldrich. Ultra-dry dimethylformamide (DMF), ultra-dry dimethyl sulfoxide (DMSO), ultra-dry chlorobenzene (CB), dry isopropanol (IPA), 4-tert-butyl pyridine, and lithium bistrifluorosulfonylimide (LiTFSI) were purchased from Sigma-Aldrich. All the chemicals are used as received without further purification. Conductive glass, fluorine-doped tin oxide (FTO) (8 Ω/sq), and titanium dioxide paste (30 NRD) were purchased from Greatcell LTD, West Perth, WA, Australia.

### 2.2. Fabrication of Solar Cells

#### 2.2.1. Substrate Preparation

FTO substrates were consecutively cleaned using a 2% Hellmanex aqueous solution, milli-Q water, and isopropanol by sonicating for 5 min for each solvent. After drying with a nitrogen gun, the samples were kept in a box.

#### 2.2.2. TiO_2_

A precursor solution of tetra ethyl ortho titanate (0.57 g) was dissolved in a mixture of ethanol and hydrochloric acid (5 + 0.1 mL), respectively. The solution was then spin-coated on FTO substrates at 2000 rpm for 15 s. Prior to spin coating, the substrates were cleaned with O_2_ plasma for 15 min. After spin coating, the wet substrates were sequentially annealed at 200 °C for 10 min and 500 °C for 30 min, respectively.

#### 2.2.3. Mesoporous TiO_2_

A precursor solution of meso-TiO_2_ was prepared by dissolving 30 NR-D paste in ethanol in a mass ratio of paste: EtOH = 1:6. The solution was then spin-coated on top of the compact layer at 3500 rpm for 15 s. The wet substrates were sequentially annealed at 200 °C for 10 min and 500 °C for 30 min, respectively.

#### 2.2.4. Preparation of the Precursor

For perovskite FAPbI_3_, two kinds of precursor solutions were used: Stoichiometric and non-stoichiometric. A stoichiometric precursor solution (1 FAI:1 PbI_2_) was prepared by dissolving a mixture of lead iodide (903.2 mg, 1.96 mmol), formamidinium iodide (336.2 mg, 1.96 mmol), and methylammonium chloride (39.04 mg) in a 1 mL mixed solution of DMF and DMSO (DMF (v):DMSO (v) = 4:1) in the glove box and was stirred using a magnetic stirrer without heating. A perovskite obtained from a stoichiometric solution is hereinafter referred to as a PVK (S).

Non-stoichiometric precursor solutions were prepared in the same manner but with a higher PbI_2_ content in relation to FAI. We introduced non-stoichiometry in the precursor in such a way that the total mass of the constituents (FAI and PbI_2_) participating in the reaction remained constant. The perovskite obtained with excess Pb and further denoted by PVK (NS) is referred to as a lead-rich perovskite, which will be justified on the basis of SEM and XPS tests.

#### 2.2.5. Perovskite Layer Fabrication

The perovskite active layer was deposited using an anti-solvent method, with ethyl acetate as the antisolvent. The perovskite precursor solution was deposited on the freshly prepared FTO/c-TiO_2_/mpTiO_2_ substrate, and a two-step spin-coating method was applied. The first step proceeded at 1000 rpm with an acceleration rate of 200 rpm/s for 5 s. The second step was followed by 6500 rpm with an acceleration rate of 2000 rpm/s for 25 s. Furthermore, 1000 µL of EA was applied at the 10th second after the spin-coating had ensued. After spin-coating, the substrate was annealed at 150 °C for 10 min to enable the formation of the black-phase FAPbI_3_.

#### 2.2.6. 2D Perovskite Fabrication

The surface 2D perovskite was fabricated by the post-treatment of the 3D perovskite with a solution of the 2D reagents, octyl ammonium iodide (OAI) in isopropanol with a concentration of 0.01 M–0.04 M.

The solutions were spin-coated on the surface of the perovskite film for 15 s at 3000 rpm. The full perovskite film was then annealed at 100 °C for 5 min. The procedure was carried out in a glovebox.

#### 2.2.7. Hole-Transporting Layer

Spiro-OMeTAD was used as the hole-transporting layer (HTL) material. First, 73.6 mg of Spiro-OMeTAD powder was dissolved in 1 mL of chlorobenzene. The solution was doped with 17 µL of LiTFSI (prepared by dissolving 520 mg of LiTFSI in 1 mL of acetonitrile) and 30 µL of 4-tertbutylpyridine, respectively. The mixed Spiro-OMeTAD solution was then spin-coated on the surface of the perovskite at 2000 rpm for 30 s.

#### 2.2.8. Top Surface Contacts

The gold electrode was thermally evaporated on the surface of the HTL with the shadow mask, with an area of 0.25 cm^2^. The thickness of the gold electrode was 80 nm, and the evaporation rate was adjusted to 0.01 nm/s for the first 10 nm and 0.08 nm/s for the rest of the procedure.

### 2.3. Characterization Techniques

#### 2.3.1. Current Voltage (I-V) Measurements

Photovoltaic performance measurements were carried out under AM1.5G standard conditions by I-V curve tracing using a Keithley 2401 source meter under simulated AM1.5G irradiation (100 mW cm^−2^). A Photo Emission Tech AAA class solar simulator calibrated against certified reference Si solar cells with a KG-3 filter (Institute Fraunhofer ISE, Breisgau, Germany) was used.

Solar cells were masked to 0.25 cm^2^. I-V measurements were performed in two scan directions, from −0.2 V to 1.5 V, which we call the forward scan, and from 1.5 V to −0.2 V, which we call the reverse scan. The scan rate was set at 100 mV/s.

#### 2.3.2. Scanning Electron Microscopy (SEM)

The topography investigations were performed using scanning electron microscopy (tabletop TM3030, Hitachi High-Tech, Tokyo, Japan) with an accelerating voltage of 10 V in the backscattered electron mode.

#### 2.3.3. UV-Vis-NIR Spectroscopy

The transmittance and reflectance of the perovskite films were measured using an optical spectrophotometer (Lambda 950S, Perkin Elmer, Waltham, MA, USA). The band gap of the perovskite films was calculated by Tauc’s plot, which uses the values of absorption coefficient α of the film calculated from the transmittance (T) and reflectance (R) data according to the formula: α = −1/d × ln[(1−R)T]. We used the reflectance data in order to increase accuracy.

#### 2.3.4. Transient Absorption Measurements (TAS)

Ultrafast dynamics were determined using a broad-band transient absorption (TA) setup (Helios spectrometer, Ultrafast Systems, and Spectra Physics laser system), described previously [25]. The IRF (pump-probe cross-correlation function) was approximately 200 fs (full width at half maximum), and transient absorption measurements were performed in the time range of up to 3 ns. One excitation wavelength was used, 490 nm, and the spectra were probed in the range of 500–830 nm. Transient absorption spectra were analyzed with Surface Xplorer 2.4.3.153 Software (Ultrafast System).

#### 2.3.5. X-ray Photoelectron Spectroscopy (XPS)

The X-ray photoelectron spectra (XPS) were recorded using the hemispherical analyzer EA 15 (PREVAC, Rogow, Poland) equipped with the dual anode X-ray source RS 40B1 (PREVAC). The measurements were performed using Al Kα (1486.6 eV) radiation and an analyzer pass energy of 100 eV. The spectra were recorded in normal emission geometry with an energy resolution of 0.9 eV. The spectrometer was calibrated with Ag, Au, and Cu foil according to ISO 15472:2010 standards [26]. An ultra-high vacuum (UHV) of 8·10^−9^ mbar was maintained during the measurements. The analyzed area was approximately 3 mm^2^, and the penetration depth was approximately 10 nm.

The perovskite samples were mounted and positioned at the dedicated holder and pumped out to a high vacuum and then transferred into the UHV chamber. The survey and high-resolution spectra were acquired for every sample. The spectra were analyzed using the analysis software CasaXPS 2.3.24PR. The electron binding energy (BE) scale was calibrated for the Fermi edge at 0.0 eV. The Shirley-type spectrum background was used. The high-resolution spectra were deconvoluted with the Voigt function (Gaussian to Lorentzian profile ratio of 70:30). The spectra were compared respectively to the background level.

#### 2.3.6. X-ray Diffraction (XRD)

The XRD measurements of the perovskite films were carried out using a Bruker D8 Discover diffractometer equipped with a Cu Kα X-ray source. Bragg–Brentano (Ɵ-2Ɵ) X-ray diffraction continuous scans were performed over the range of 2Ɵ = 10–100° at 2 s per step with a step size of 0.02°. The phase composition of the perovskite films was analyzed using Diffrac.EVA v.3.0 software with the ICDD PDF-4+ crystallographic database.

#### 2.3.7. Atomic Force Microscopy (AFM)

Kelvin Probe Force Microscopy (KPFM) experiments for the standard perovskite solar half-cells at room temperature (22 °C) and humidity below 44% were performed using the Dimension3100 setup with the NanoscopeV controller. The KPFM mode was used in order to determine the electrical response on the top of 3D and 2D perovskite layers at the microscale. To obtain information related to the surface potential, a tip (Type: Arrow™ EFM) with PtIr thin layer was used. The measurement was performed in two-pass, lift mode (that is, the tip of the AFM was approximately 120 nm above the surface of perovskite solar half-cells) in air. The obtained data were processed using Gwyddion software 2.60 [27]. KPFM measurements in the dark and under irradiation were conducted. For the illumination of working perovskite solar half-cells, the following light source was used: A white cold LED COB with electrical power of 10 W, a viewing angle of 140°, a color temperature of 6500 K, and a luminosity of 850 lm. In Figure S1, a schematic representation of a KPFM measurement setup is shown (see Supporting Information).

In order to understand the significant effect on electrical properties at the microscale of a 2D perovskite (PVK) layer spread on top of a 3D PVK, atomic force microscopy studies have been carried out in the dark and under light conditions. Based on Kelvin probe force microscopy (KPFM) experiments, changes in the surface potential (SP) in different light conditions and for stoichiometric and lead-rich perovskite 3D PVK with and without 2D passivation layers have been estimated.

## 3. Results and Discussion

### 3.1. Obtaining the Right Precursor Stoichiometry for Obtaining High-Efficiency Solar Cells

As a starting point, we fabricated 3D FAPbI_3_ solar cells by combining 1 molar mass (1M) of the constituents. The reaction leading to the formation of the 3D perovskite can be written as follows:

1M FAI + 1M PbI_2_ → 1M FAPbI_3_ (1)

**Figure 1:**
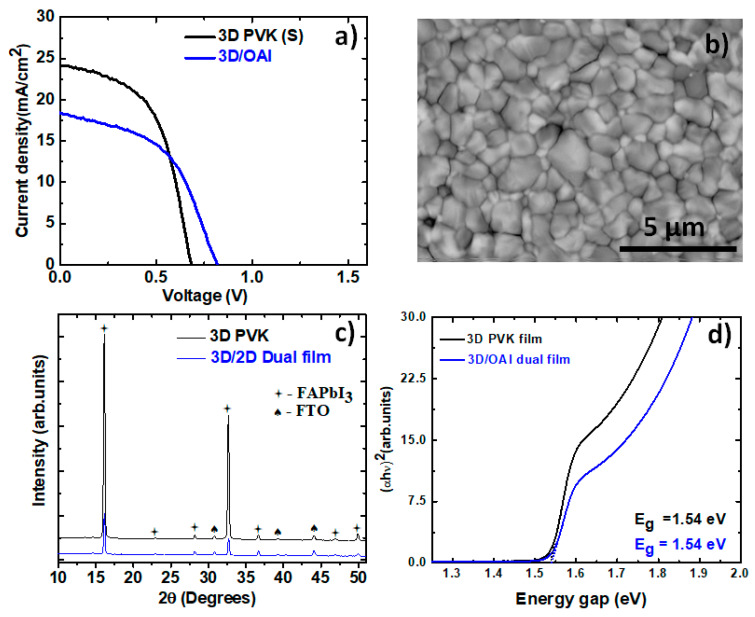
Current–voltage (I-V) characteristics for the FAPbI_3_ perovskite PSCs from stoichiometric solution with and without OAI (**a**); SEM Image showing the topography of the FAPbI_3_ perovskite films from stoichiometric solution (**b**); X-ray diffractograms (XRD) of FAPbI_3_ perovskite films from stoichiometric solution with and without OAI (**c**); Tauc’s plot showing the band gap of the FAPbI_3_ perovskite films from stoichiometric solution with and without OAI (**d**).

The 3D perovskite films were processed following the optimized procedure explained in the previous section. The solar cells were fabricated in the n-i-p configuration. The following sequence of layers was used: Glass-fluorine doped tin oxide (FTO)/compact titanium oxide (c-TiO_2_)/mesoporous (m-TiO_2_)/3D-FAPbI_3_ perovskite/2D-perovskite/spiro-OMeTAD/Au. The current–voltage characteristics of the corresponding PSCs (the best cells) are shown in Figure 1a. The solar cells prepared with stoichiometric precursors show a PCE of 9.6%, which is very different from many published works. In order to prevent the formation of the δ phase, we deposited a thin film of 2D perovskite made with OAI (10 mM dissolved in 1 mL IPA) on the surface of the 3D perovskite film. Even after modifying the interface, the PCE of the solar cell did not improve but rather degraded to 7.5%. The photovoltaic parameters of the solar cells are shown in Table 1.

When we compare the photovoltaic performance of the two solar cells, the PSC made with the 3D/OAI film appears more resistive, resulting in a decreased J_sc_ and FF. We suspect that this resistive behavior comes from the addition of OAI to the surface of the film. The insulating nature of the long alkyl chain could have contributed additional series resistance to the solar cell, thereby decreasing its efficiency. This suggests that a 2D perovskite film may not have formed on the surface of FAPbI_3_ as per expectations, and instead, we merely deposited a thin layer of OAI and partially insulated the film’s surface.

In Figure 1b, we show the morphology of the bare perovskite film made with the stoichiometric precursor. The perovskite film has a dense microstructure with large, pinhole-free grains on the surface. The presence of the cubic α phase is confirmed by the XRD pattern of the perovskite film shown in Figure 1c. The characteristic peaks of the black phase have been indexed using the PDF card 00-069-0999 from the ICDD PDF 4 database. Apart from FAPbI_3_, there is a very small amount of PbI_2_ in the film, as indicated by the peaks at 14.72° and 45.2° (indexed by the PDF card no: 00-007-0235) and reflections from the back contact, FTO (PDF card no: 04-003-0649). Both the bare perovskite film and the film with OAI exhibit the same diffraction pattern, but the intensity of the FAPbI_3_ peaks in the 3D/OAI film is diminished considerably. We attribute this effect to the presence of a thin amorphous layer at the surface formed by OAI. We calculated the band gap of the perovskite films using Tauc’s plot, which shows a sharp absorption onset around 800 nm, corresponding to a band gap of approximately 1.54 eV for both films (Figure 1d).

In order to gain more understanding of the 3D perovskite (S) films and possible causes responsible for the poor photovoltaic performance of the PSCs fabricated with the perovskite film 3D PVK (S) and 3D/OAI, we tested the films using KPFM. To begin with, using KPFM, we can measure the surface potential (SP) of the films in the dark and under illumination. The difference in the values of SP between dark and illumination gives us the surface photovoltage (SPV) generated in the perovskite half-solar cells. In this scenario, the terminology ‘perovskite half-solar cell’ is analogous to ‘perovskite film’ since the film contains one charge transport layer and one electrode. The generation of (SPV) can be correlated with the band bending taking place in the PSCs under illumination. The results of the KPFM measurements are shown in Figure S2 (Supporting Information). In Table S1, we summarize the information obtained from AFM and KPFM measurements.

The small fluctuations in the obtained values of SP are expected phenomena and are related to the intensity of the light source used, the exact area of illumination, and the architecture of a perovskite half-solar cell. In our case, the perovskite solar semi-cells were illuminated from above and from the side of the 3D/2D active layer and not from the semi-transparent conductive electrode as shown in Figure S1. This leads to small changes in the SP values between dark and light conditions [28]. Moreover, it is important to keep in mind that the measurements have been carried out on perovskite films without the HTL. In the absence of the HTL, there is less efficient charge separation, which is reflected in weaker SP fluctuations between dark and light measurements. Fluctuations in the measured values of the work function from the top of such a thin film stack, which indicate the absence of a charge transporting layer, which leads to a reduction in the difference of potential between dark and light experiments [29,30].

For the perovskite 3D film from stoichiometric solution PVK (S) without OAI, an increase in SP after illumination was observed. In contrast, the SP decreases under illumination when an organic layer is applied. This could imply the presence of an insulating component, which restricts the band bending in the photoactive layer.

The empirical formula for the most common 2D (Ruddlesden-Popper) perovskites is given by the empirical formula, (LC)_2_(SC)*_n_*_−1_Pb*_n_*I_3*n*+1_ where LC is a large cation such as OA^+^ and SC is a small cation such as FA^+^, and *n* is the number of (PbI_6_)^4−^octahedral units sandwiched between the large cations. The lowest value *n* can have is 1, which corresponds to a pure 2D perovskite with the formula (OAI)_2_PbI_4_. This means that in order to form a 2D perovskite layer, we need PbI_2_ along with OAI. Under stoichiometric conditions, the reaction leading to the formation of the FAPbI_3_ black phase after spin coating and annealing will consume all the available PbI_2_ in the precursor, leaving nothing behind. Therefore, when using a stoichiometric composition, there is no additional PbI_2_ to form a 2D perovskite layer, and depositing OAI on the surface of the 3D film only forms a thin insulating film, which degrades the solar cell’s performance even further.

In order to have additional PbI_2_ to form a 2D perovskite layer, we intentionally make the perovskite precursors non-stoichiometric. The composition of non-stoichiometric precursors is (1 − x) FAI:(1 + x) PbI_2_ where the amount of ‘x’ is varied between 0.05 and 0.15. We then fabricated PSCs as before. The spread of the PCEs in the PSCs made with different x values along with the IV curve of the best cell is shown in Figure S3 (Supporting Information). We observe the highest efficiency of 17% for the condition x = 0.1. All the photovoltaic parameters (J_sc_, V_oc−_, and FF) show a significant improvement compared to the PSC from the stoichiometric solution.

**Figure 2:**
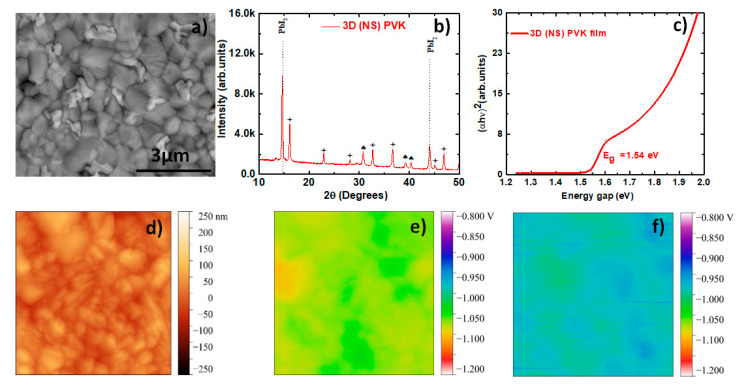
SEM Image showing the topography of the FAPbI_3_ perovskite film from non-stoichiometric solution (NS) PVK (**a**); X-ray diffractograms (XRD) of the (NS) PVK (**b**); Tauc’s plot showing the band gap of (NS) PVK (**c**); topography of 3D (NS) perovskite active layer without OAI obtained by atomic force microscopy (**d**); surface potential measured by Kelvin probe force microscopy in the dark (**e**) and under white light on the top of 3D (NS) perovskite active layer (**f**).

In Figure 2a, we show the morphology of the perovskite film embodying the best solar cell. Similar to the perovskite film fabricated from a stoichiometric solution, the morphology of the perovskite film made from the non-stoichiometric solution is also dense and compact. Along with the big grains of perovskite, we can also see small, bright particles scattered on the surface. These bright particles can be associated with unreacted PbI_2_ (as elements with heavier atomic numbers appear brighter in the backscatter electron detection mode). Similar examples can be found in several other papers, which discuss 3D perovskites [31,32]. The crystallographic features of the Pb-rich perovskite film are shown in Figure 2b. Similar to the perovskite film made from the stoichiometric solution, the reflections in the XRD pattern of this film are also matched by FAPbI_3_, PbI_2_, and FTO using the same references. However, the main difference in this XRD pattern comes from the intensity of PbI_2_. Due to the presence of excess PbI_2_ present in the films, the intensity of the peak at 14.6° is very high in this film. From Tauc’s plot shown in Figure 2c, we calculate the band gap of the film to be 1.54 eV, which is the same as the stoichiometric perovskite film, suggesting that small changes induced in the stoichiometry of the precursor do not influence the band gap of the absorber.

In Figure 2d–f, we show the topography (d) and SP measured in the dark (e) and under white light (f) for the 3D Pb-rich perovskite PVK (NS) half solar cell. An increase in SP after illumination is observed compared to the SP measured in the dark. This implies the generation of SPV in the perovskite half solar cell, which leads to changes in the work function [30]. We also notice that the magnitude of SP in the PVK (NS) film is higher when compared to the SP of the film from the stoichiometric solution PVK (S). Comparing the photovoltaic performance of the PSC made from different precursors, we are inclined to believe that perovskite films made under Pb-rich conditions are less defective or more defect-tolerant than the stoichiometric films. A higher SPV in the PVK (NS) films suggests optimal band bending at the perovskite/HTL interface, leading to the efficient transport of carriers at the interface and better photovoltaic performance.

At this point, we are still unable to answer why the photovoltaic performance of the PSCs fabricated using a stoichiometric precursor has poor efficiency. From the results we have thus far, we observe that the bulk properties of the perovskite films (microstructure, crystallinity, and band gap) are essentially the same irrespective of the precursor composition. In the literature, we often find a Pb excess composition being used for high-efficiency solar cells. There are many reports that support this claim [33,34,35]. Our results also favor the same observation. However, in order to look for specific differences among the perovskite films that could explain the photovoltaic performance of the PSCs presented till now, we studied the surface of the bare perovskite films using XPS.

With XPS, we measured the total concentration of the elements at the sample surface (Survey spectra shown in Figure S4) and the deconvoluted absolute spectrum of every element measured in the survey spectra. Table S2 (Supporting Information) shows the percentage of all the elements measured in the survey spectra. The XPS survey spectra are sensitive to the surface composition of the perovskite films. With the given beam energy, information about the composition is obtained from the first 5–10 nm of the surface. Since the films differ from each other in the amount of PbI_2_, we first looked at the differences in the Pb 4f spectra and I 3d from the survey spectra for the different perovskite films.

The film ‘3D PVK (NS)’ made from a non-stoichiometric precursor is Pb rich on the surface due to the presence of excess PbI_2_ in the film in comparison with both stoichiometric films. The increase in the I signal for the 3D/OAI film is due to the presence of OAI at the surface of the film. In the same film, we observe small concentrations of O_2_ and N_2_ (shown in Table S2 in Supporting Information) at the surface. This is due to the hydrophobic nature of the long alkyl chain in OAI. The amount of Iodine measured from the surface of 3D PVK (S) is higher than that of sample 3D PVK (NS). The total mass of Iodides taking part in the formation reaction of the perovskite is the same in all three films. Therefore, this observation suggests that changing the precursor composition could actually result in perovskite films with different surface compositions or, in other words, different ways in which the surface of the FAPbI_3_ perovskite film terminates.

We analyzed the absolute spectra of each element in the survey spectra in detail. The chemical states are assigned to certain peaks according to the databases [36,37]. For this discussion, we use the results from the absolute spectra of Carbon (C), Nitrogen (N), and Oxygen (O).

When we deconvolute the absolute 1s spectra of carbon (C) as shown in Figure S6 (Supporting Information), we see major differences in the peak at 286.5 eV. This peak corresponds to the bonding of C with Nitrogen (N) in the form of a C-N single bond. This bond is characteristic of amine (C-NH_2_) or amide (NH_2_-C=O) groups. Amide groups are present in the precursor solvent DMF. DMF has a boiling point of 153 °C. We use DMSO alongside DMF in a 4:1 ratio. DMSO has a boiling point of 189 °C. The annealing temperature of the perovskite film is 150 °C. During the annealing step, rapid crystallization of the perovskite takes place, forming dense grains within the first few seconds before all of the solvents present in the wet film could fully evaporate. DMF offers strong polarity and strong intermolecular forces, which makes solvent evaporation a difficult task. As a result, it is very likely that a fraction of the solvent present in the wet film is trapped between the grains. Residues of DMF and DMSO are regularly present in perovskite films after conventional annealing [38,39,40,41]. Therefore, the primary contribution of C=O in these films is expected to come from residual DMF present in the film after annealing. In Table 2 below, we show the proportion of C=O bonds calculated from both C 1s spectra and O 1s spectra.

The amount of C=O bonds is almost the same in both perovskite films from stoichiometric PVK (S) and non-stoichiometric solution PVK (NS) without the OAI layer. In the film 3D/2D PVK (S), the % of C=O is lower than in the other two films for two reasons: One is due to the presence of OAI, which covers the surface of the 3D perovskite film, limiting the depth of penetration of the probing beam, and the second is due to the low amount of O_2_ adsorbed on the surface of the film, as seen in Table 2. The C-NH_2_ single bond is present in both FAI and DMF. However, the difference in the peak proportions among the analyzed samples indicates that they could be influenced by the presence of FA^+^ ions on the surface of the perovskite films. It is possible for MA^+^ ions to contribute to this bonding because the precursor utilizes 35% MACl in the mixture. The fact that our absorbers exhibit a band gap of 1.54 eV as opposed to the widely reported 1.47 eV forces us to think that some MA^+^ could be substituted for FA^+^, leading to a widening of the band gap. We believe that the adsorption of N_2_ from the atmosphere did not contribute to this bond because the total amount of N_2_ measured from the surface of the lead-rich perovskite film is lesser than that of the stoichiometric film.

We make the non-stoichiometric perovskite precursor FAI deficient intentionally. It contains approximately 12 wt% FAI less compared to the stoichiometric film. In spite of this considerable difference, the surface of the bare perovskite film is rich in C-N signature bonds (Table 3), which suggests that the surface of this perovskite film is possibly terminated by FA^+^ ions. Referring to the work of Oner et al. [42], we find that in FAPbI_3_ films prepared under Pb-rich conditions, surface termination of the bulk FAPbI_3_ by FA^+^ ions is very much a possibility. It is the most suitable surface considering the formation energies of defects and the surface energy. The FA^+^-terminated surface has the lowest surface energy and, therefore, offers the most stable surface of all the possibilities. For the same criteria, I^−^ termination is found to be the most vulnerable surface against all types of defect formations, as well as one of the most energetically unfavorable surfaces. On the I^−^ terminated surface, the formation energies of the possible defects are quite low, and hence they can be created easily after the film has formed. Such defects are likely to create deep transition levels in the band gap, thereby possibly degrading the photovoltaic performance.

Taking into account the results from the survey spectra, we see that a perovskite film obtained from a stoichiometric solution, PVK (S), is I^−^ rich in comparison and would likely result in an I^−^ terminated surface. An over-stoichiometric amount of PbI_2_ would result in a Pb-rich surface due to excess PbI_2_ that precipitates at the end of the reaction, but the resulting perovskite film is possibly surface terminated by FA^+^ ions. From our findings, it is clear that the precursor stoichiometry heavily influences the surface composition of the perovskite and thereby the nature of the surface termination, which in turn affects the performance of the solar cells.

### 3.2. Optimization of the Concentration of the Large Cation to Improve the PCE

In order to ensure a stable solar cell over a long period of time, it is necessary to preserve the α phase and prevent the phase transition of FAPbI_3_. Therefore, we deposited OAI on the surface of the lead-rich perovskite film.

**Figure 3:**
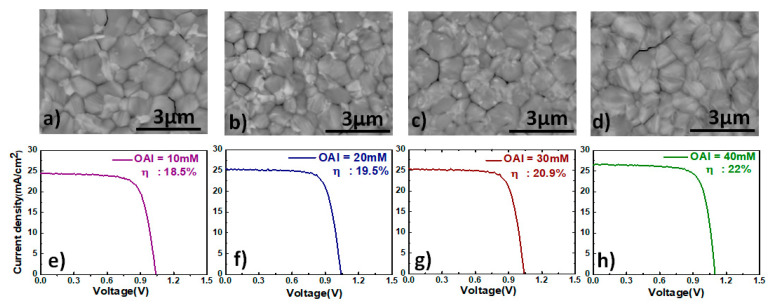
SEM images showing the topography of lead-rich FAPbI_3_ perovskite films with varying concentrations of OAI (**a**–**d**); Current density–voltage (IV) characteristics for the lead-rich FAPbI_3_ perovskite films with varying concentrations of OAI (**e**–**h**).

We varied the concentration of OAI from 10 mM to 40 mM, and by doing so, we varied the thickness of the 2D perovskite film, as the film thickness strongly depends on the concentration of the large cation. In Figure 3a–d, we show the morphology of the perovskite films containing different thicknesses of the OAI layer. In comparison with bare lead-rich perovskite film without the 2D layer, the perovskite films containing the 2D layer show an improvement in the grain size and display a more uniform morphology. When the surface of FAPbI_3_ is treated with a solution of IPA containing OAI and subsequently annealed, recrystallization of the 3D film takes place, resulting in more uniformly shaped grains [43,44]. We also notice that as the concentration of OAI increases, the amount of PbI_2_ present on the surface of the film decreases gradually (fewer bright particles), and when the concentration of OAI reaches 40 mM, we no longer observe the bright PbI_2_ particles in the image. At a concentration of 40 mM, more OAI is available to react with the excess PbI_2_ present on the surface, thereby forming a thick 2D perovskite layer on top of the 3D perovskite film. We also notice that perovskite films containing thicker 2D perovskite layers appear brighter than films without the 2D layer. This is because the 2D perovskite has lower conductivity than PbI_2_ and FAPbI_3_, and as a result, charge accumulation takes place at the surface, making it look relatively bright [45].

In Figure 3e–h, we show the JV curves in the forward scan for the corresponding champion PSCs. The PSCs with the 2D perovskite layer display an improvement in the PCE in comparison with the PSCs made without the 2D layer, primarily due to the increase in the open circuit voltage of the devices. The formation of a thin layer of 2D perovskite at the surface acts as a capping layer, protecting the film from ambient conditions. Additionally, the ammonium atoms in OAI are capable of filling in for defects [46,47] on the surface of the film, thereby minimizing the recombination losses in the absorber. As a result, the V_oc_ of the PSC’s increases contributed to an increase in the overall PCE. The consumption of uncoordinated PbI_2_ from the surface by the large cation also results in the formation of a graded 3D/2D heterojunction, which exhibits a different energy level at the interface and constructs a built-in electric field (V_bi_), facilitating charge extraction [48], with optimal band alignment facilitating carrier transport across the junction, resulting in an improved fill factor.

In Figure S8, we show the topography of the 3D/2D perovskite films along with the color plots showing the distribution of the SP from KPFM measurements. We estimated the SPV for the films containing OAI by taking into account the SP measured in the dark and under illumination. The values of the SPV for the non-stoichiometric perovskite film containing different concentrations of OAI (10 mM, 20 mM, 30 mM, and 40 mM) are 25 mV, 57 mV, 30 mV, and 13 mV, respectively. Comparing the SPV of the control sample (without OAI, which is 85 mV) with that of the perovskite films with the OAI layer, it is quite clear that the SPV decreases with an increase in the concentration of OAI, or in other words, with the formation of a 2D perovskite at the interface. A similar observation was reported in the work of Zhang et al. [30]. Taking this published work into consideration, we can postulate that with the incorporation of OAI, there is a formation of a thin 2D perovskite layer on the surface, which minimizes the density of the acceptor trapping states localized on the surface of 3D perovskite active layer. This possibly causes a decrease in the magnitude of the trap states on the surface [30], which leads to an improvement in the photovoltaic performance of the PSCs with the 2D perovskite layer. Overall, from KPFM, we predict a reduction of defects at the surface of the film with the formation of a 2D perovskite film. This finding is consistent with the improvement in the IV parameters of the PSCs.

**Figure 4:**
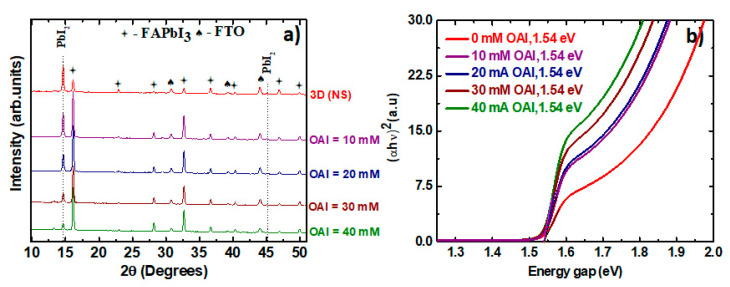
X-ray diffractograms (XRD) of lead-rich FAPbI_3_ perovskite films without OAI and with varying concentrations of OAI (**a**); Tauc’s plot for extrapolating the band gap of the lead-rich perovskite films without OAI and with varying concentrations of OAI (**b**).

In Figure 4a, we show the XRD patterns of the perovskite films with different thicknesses of the 2D perovskite layer along with the lead-rich film without the 2D perovskite layer. As shown previously, the α phase is present in all the films. As the concentration of the large cation is increased from 10 mM, we see that the intensity of the main PbI_2_ peak starts to decrease, reaching a bare minimum for a concentration of 40 mM. This indicates the consumption of PbI_2_ with increasing OAI concentration, thereby forming thicker 2D perovskite layers, as we pointed out in Figure 3. The decrease in the intensity of PbI_2_ is accompanied by an increase in the intensity of the perovskite peak at 16.2°. This is due to the formation of a more ordered surface as a result of the recrystallization of the 3D film during the surface OAI treatment.

**Figure 5:**
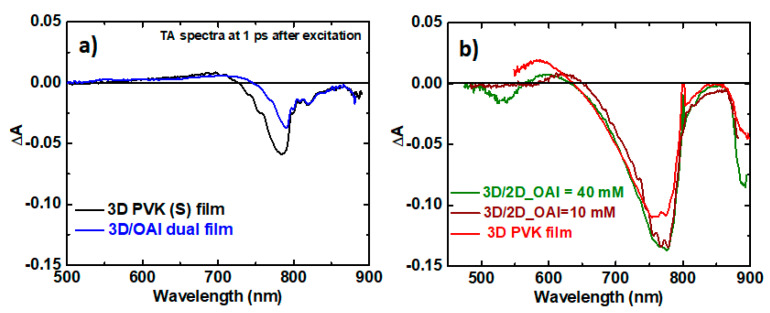
Transient absorbance (TA) spectra of perovskite film PVK (S) with and without OAI (**a**); TA spectra of perovskite film PVK (NS) with varying concentrations of OAI (**b**).

The band gap of the perovskite films was calculated from Tauc’s plot shown in Figure 5b. In the presence of a 2D perovskite, the absorption of photons in the measured wavelength range has considerably increased. Part of this enhancement comes from the absorption of 2D perovskite, which has a band gap in the region of approximately 2.4 eV [49] as opposed to the band gap of the FAPbI_3_, which has a band gap of 1.54 eV in all cases. However, this increase in absorption does not necessarily translate to an increase in the J_sc_ of the solar cells, as one would expect. Only for the perovskite films that contain thick 2D layers (30 mM and 40 mM) do the resulting solar cells show an increase in J_sc_ of approximately 1 mA/cm^2^ compared to the rest of the solar cells. The presence of 2D layers does not change the band gap of the 3D FAPbI_3_ perovskite films; rather, the 2D perovskite merely increases the absorbance in the high-energy region of the spectrum. Such phenomena have also been previously reported [50].

We performed a detailed optical analysis on all the perovskite films discussed so far using TAS. We first show the TAS spectra of the stoichiometric perovskite film and the film post-treated with OAI in Figure 5. The measurements were performed with both the probe and pump exciting beams entering from the perovskite side. The spectra for both films show a photobleaching peak around 780 nm, which corresponds to the black phase. For the n = 1 two-dimensional perovskites made with OAI, PB peaks are expected to be seen around 506 nm (2.45 eV) [49]. In the case of the OAI-treated film, there are no PB peaks in this region, which confirms the absence of any 2D perovskite in the film. In the set of lead-rich perovskite films, only an OAI concentration of 40 mM was bleached at 540 nm observed, as can be seen in Figure 5b. For the reference lead-rich film without OAI, there is a positive transient absorption signal in this spectral range instead (originating from the 3D phase), while for an OAI concentration of 10 mM, we can observe a signal in between, around zero, which might indicate the small presence of a 2D phase but without clear bleaching. For two other OAI concentrations (20 and 30 mM), we were not able to measure reliable TAS spectra below 600 nm, likely due to the too-high stationary absorbance there and/or too-low concentration of the 2D phase. FAPbI_3_ has a very high optical absorption in the region of 550–800 nm as seen from Tauc’s plot, and combined with the high thickness of the layer (approximately 750 nm as shown in the color-marked cross-section TEM image in Figure S9), most of the output signal was dominated by FAPbI_3_, and it was not possible to obtain any signal from the thin layer of 2D perovskite, which has ground-state bleaching in the low-energy part of the spectrum. Only when the thickness of the 2D phase was considerably higher (as in 40 mM PVK film), the ground-state bleaching could be recorded. We expect this signal to come from the low-dimensional n = 1, 2D perovskite [49]. However, the position of this bleachinig is shifted to a higher wavelength as opposed to the band gap of n = 1, 2D perovskite, which is approximately 506 nm (2.45 eV). This indicates that the signal could originate from higher-order 2D perovskites (like n = 2). When we look back at the formula for 2D perovskites mentioned earlier in the manuscript, it is clear that small cations such as FA^+^ or MA^+^ are needed to form higher-order perovskites such as n = 2, 3, etc. In the lead-rich perovskite films, we have seen that the surface is terminated by FA^+^ ions, as evidenced by XPS. During the annealing step of the post-treatment with OAI, it is very likely that some of these FA^+^ ions on the surface would have reacted with OAI and PbI_2_, forming a higher-order 2D perovskite such as (OA)_2_FAPb_2_I_7._ The band gap of 2D perovskites increases with increasing n. Therefore, a band gap of 540 nm (2.3 eV) would correspond to a 2D perovskite with n = 2 [49]. The formation of such higher-order 2D perovskites at the interface of 3D FAPbI_3_ has been reported previously [16]. The overall optical analysis by TAS correlated with the published literature reveals the exact composition of the 2D perovskite layer formed during the post-treatment with OAI.

We share more information on the dynamics of the charge carriers by comparing the TA response (∆A) of the photo-bleached peaks as a function of the pump-probe delay time (Figure S10). We probe the kinetics at 550 nm (within the 2D phase bleach band) and around 780 nm (3D phase bleach). For stoichiometric samples, no negative amplitude due to 2D phases can be seen (Figure S10c), and the kinetics of the bulk bleaching are similar for the samples with and without OAI (Figure S10a). In contrast, for the lead-rich sample with an OAI concentration of 40 mM, we can clearly see 2D-phase bleaching (negative ∆A) kinetics that decay on the time scale of single and tens of ps (Figure S10d). The decay may be due to the direct hole transfer from the 2D phase to spiro-OMeTAD and/or energy or the electron transfer to the 3D phase (e.g., [32]). The kinetics at 550 nm of the sample with 10 mM of OAI are approximately zero, which might be due to the overlap of the positive signal of the 3D phase (the sample without OAI) and the negative contribution of 2D phases (Figure S10d). However, likely more convincing proof of the presence of the 2D phase comes from the analysis of 3D phase kinetics (at 775 nm) of non-stoichiometric samples (Figure S10b). We can expect a delayed partial population of the 3D phase from the 2D phase, and this is indeed what is seen for the samples with 10 mM and 40 mM of OAI: The bleaching amplitude becomes larger, and the minimum is shifted towards longer times with respect to the reference lead-rich film without OAI.

## 4. Conclusions

We have successfully fabricated highly efficient FAPbI_3_ PSCs with the best PCE of 22% on glass substrates. The main steps behind the fabrication of the perovskite involve adjusting the stoichiometry of the precursors (FAI and PbI_2_), followed by optimization of the concentration of the large cation, which is needed to form a 2D perovskite layer. The primary difference between a stoichiometric perovskite film and a Pb^+^ excess in lead-rich perovskite film is not strictly limited only to the amount of excess PbI_2_ present in the films at the end of annealing but also extends to the nature of surface termination in these films. A Pb^+^ excess composition offers a stable and defect-tolerant surface, which eventually results in improved photovoltaic performance of the PSCs compared to a stoichiometric composition. However, the presence of uncoordinated PbI_2_ on the surface of the films limits the PCE considerably. Performing a post-surface treatment with OAI on the lead-rich perovskite films allows OAI to react with the excess PbI_2_ already present in the film to form a 2D perovskite layer on the surface. The post-treatment also results in the recrystallization of the 3D-FAPbI_3_ perovskite surface. The 3D/2D perovskite heterojunction in the post-treated films passivates the perovskite surface and enhances the optical absorption and crystallinity of the perovskite films. All these effects synergistically work together and result in an improvement in the PCE of the respective solar cells, with maximum values reached for an OAI concentration of 40 mM.

## Figures and Tables

**Table 1:** The electrical parameters of FAPbI_3_ PSCs fabricated using a stoichiometric precursor with and without a layer of OAI.

Sample Type	J_sc_ (mA/cm^2^)	V_oc_ (V)	FF (%)	PCE (Average), Best (%)
3D PVK	22.3	0.74	58	(8.8 ± 0.4), 9.6
3D/OAI	18.4	0.82	50	(7.0 ± 0.4), 7.5

**Table 2:** The percentage of C=O bonds from the absolute spectra of C and O for the different perovskite films.

Sample	C=O %(C 1s) Absolute Spectra	C=O %(O 1s) Absolute Spectra	Sum C=O (%)
3D PVK (S)	15	8.64	23.64
3D/OAI	14.2	5.78	19.98
3D PVK (NS)	11.78	11.56	23.34

**Table 3:** Table showing the percentage of C-N bonds from the absolute spectra of C and N for the different perovskite films.

Sample	C-N (C 1S)% Absolute Spectra	C-N (N 1S)% Absolute Spectra	Sum (C-N) %
3D PVK (S)	11.7	73.7	85.4
3D/OAI	12.3	81	93.3
3D PVK (NS)	18.8	79	97.8

## Data Availability

Data are contained within the article or Supplementary Materials.

## Supplementary Materials

The following supporting information can be downloaded at: https://www.mdpi.com/article/10.3390/ma16155352/s1, Figure S1: Schematic representation of KPFM measurement setup; Figure S2: Topography and surface potential measured by atomic force microscopy methods; Table S1: Average surface roughness measured from AFM images and surface potential values of the perovskite films 3D PVK (S) and 3D/OAI; Figure S3: (a) Box plot showing the distribution of PCEs and (b) current–voltage characteristics of the best PSC made with a non-stoichiometry of 0.1; Figure S4: Survey spectra measured by XPS; Table S2: Table showing the percentage of elements from the surface of the different the perovskites measured by the XPS survey spectra; Figure S5: Absolute and deconvoluted spectra of the core level of C (1s) measured by XPS; Table S3: Table showing the proportion of the different bonds formed by C with other elements from the absolute spectra; Figure S6: Absolute and deconvoluted spectra of the core level of O (1s) measured by XPS; Table S4: Table showing the proportion of the different bonds formed by O with other elements from the absolute spectra; Figure S7: Absolute and deconvoluted spectra of core level of N (1s) measured by XPS; Table S5: Table showing the proportion of the different bonds formed by N with other elements from the absolute spectra; Figure S8: Topography of the perovskite films prepared from a non-stoichiometric precursor measured by atomic force microscopy and distribution of surface potential measured from the surface; Table S6: Average surface roughness measured from AFM images and surface potential values of the perovskite films PVK (NS) with different concentrations of OAI measured in dark and under white-light illumination; Figure S9: Color-marked cross-section TEM image of the best solar cell made with an OAI concentration of 40 mM, showing the thickness of all the layers in the stack; Figure S10: Kinetics of the transient absorption spectra measured for FAPbI_3_ perovskite films from stoichiometric solution PVK (S), with and without OAI corresponding to the bleach at 790 nm (a); FAPbI_3_ perovskite films from non- stoichiometric solution PVK (NS) with varying concentrations of OAI corresponding to the bleach at 775 nm (b); FAPbI_3_ perovskite films PVK (S), with and without OAI corresponding to the bleach at 550 nm (c); FAPbI_3_ perovskite films PVK (NS) with varying concentrations of OAI corresponding to the bleach at 550 nm (d).
