# Supplementary material for: Synergistic Effect of Precursor and Interface Engineering Enables High Efficiencies in FAPbI3 Perovskite Solar Cells
Source: Materials (Basel). 2023 Jul 30;16(15):5352. doi: 10.3390/ma16155352 (PMC10419934; doi:10.3390/ma16155352)
Supplement: Supplementary file 1 [file materials-16-05352-s001.zip › materials-2520022-supplementary.pdf]

Supplementary Materials

Synergistic effect of precursor and interface engineering enables high efficiencies in $FAPbI_3$ perovskite solar cells

Sylvester Sahayaraj [1,2], Zbigniew Starowicz [1], Marcin Ziółek [3], Robert Socha [2], Łukasz Major [1], Anna Góral [1], Katarzyna Gawlińska-Nęcek [1], Marcin Palewicz [4], Andrzej Sikora [4], Tomasz Piasecki [4], Teodor Gotszalk [4] and Marek Lipiński [1*]

[1] *Institute of Metallurgy and Materials Science, Polish Academy of Sciences, 25 Reymonta St. 30-059 Krakow, Poland.*

[2] *Centrum Badań i Rozwoju Technologii dla Przemysłu S.A., ul. Ludwika Waryńskiego 3A, 00-645 Warszawa, Poland.*

[3] *Faculty of Physics, Adam Mickiewicz University, Uniwersytetu Poznańskiego 2, 61-614 Poznan, Poland*

[4] *Department of Nanometrology at the Faculty of Electronics, Photonics and Microsystems, Wrocław University of Science and Technology, 11/17 Janiszewskiego St. 50-372 Wroclaw, Poland.*

[*]Corresponding author: Dr. Hab. Marek Lipinski

E-mail: lipinski.m@imim.pl

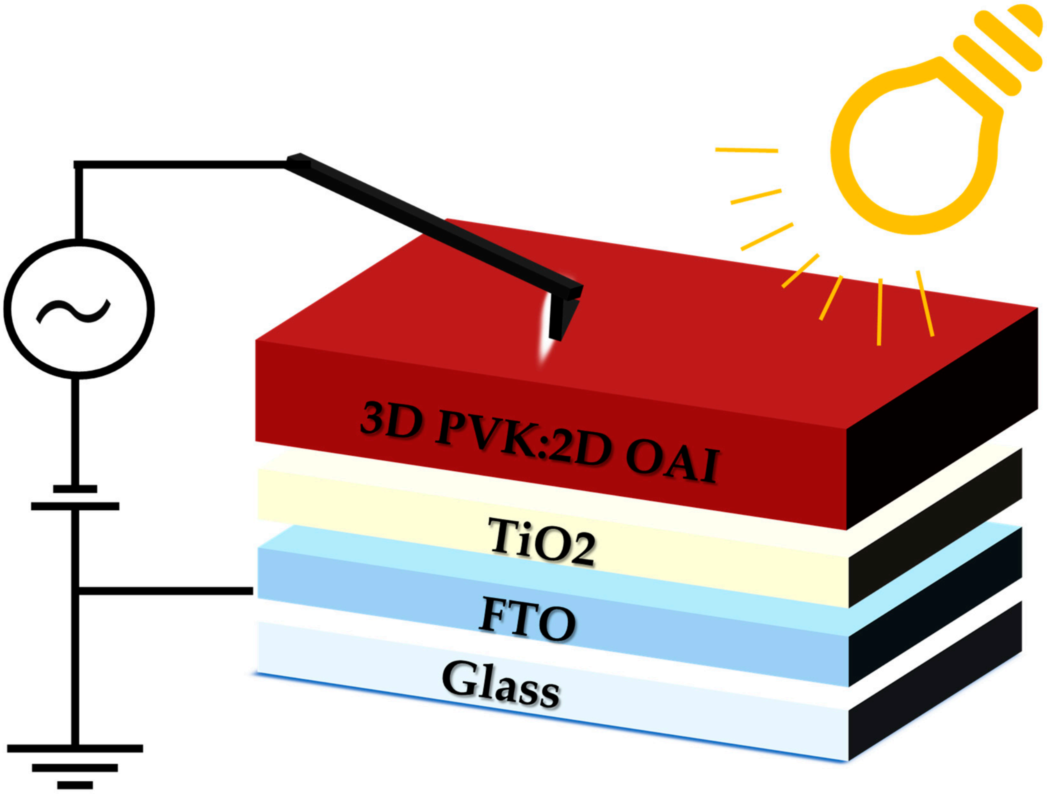

Figure S1. Schematic representation of KPFM measurement setup.

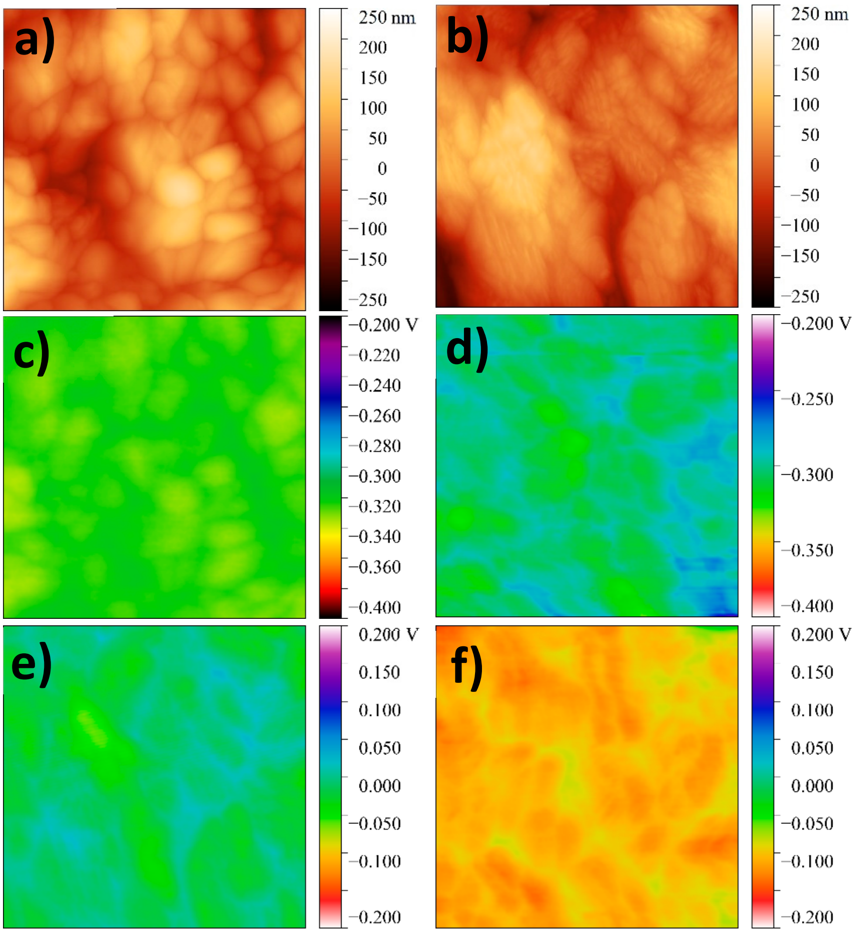

Figure S2. Topography of the perovskite films 3D PVK (S) (a) and (3D/OAI (b) measured by atomic force microscopy; color plots showing the distribution of surface potential measured from the top of the perovskite films 3D PVK (S) (c, d) and (3D/OAI) (e,f). The measurements were taken in the dark (c, e) and under light (d, f).

Table S1. Average surface roughness measured from AFM images and surface potential values of the perovskite films 3D PVK (S) and 3D/OAI measured in the dark and under white-light illumination.

**Sample**	**Sq**	**Average value of SP**	**Average value SP at white light**
	[nm]	[V]	[V]
**3D PVK (S)**	56.2	-0.32525	-0.3021
**3D/OAI**	58.5	-0.01249	-0.1081

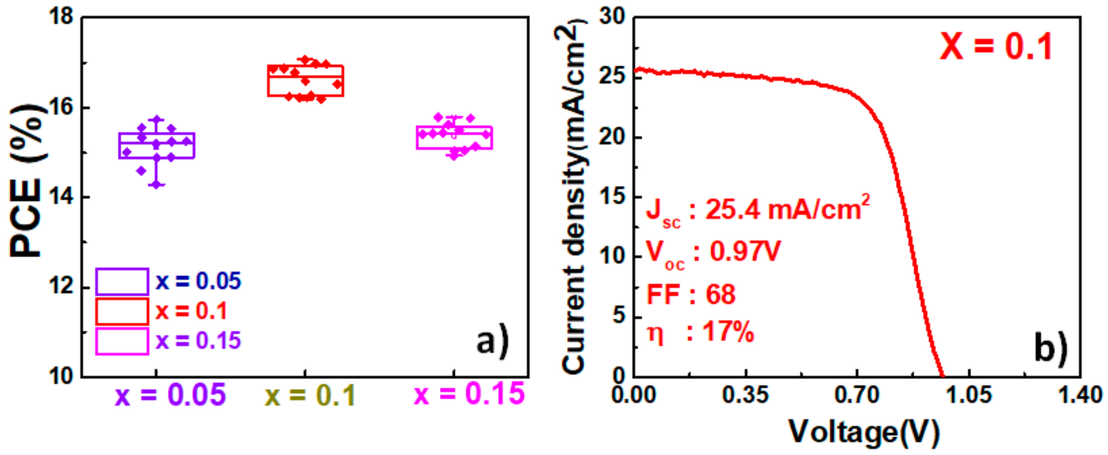

Figure S3. Box plot showing the distribution of PCEs measured for PSCs with different magnitudes of non-stoichiometry indicated as x (a); current–voltage characteristics of the best PSC made with non-stoichiometry of 0.1 (b).

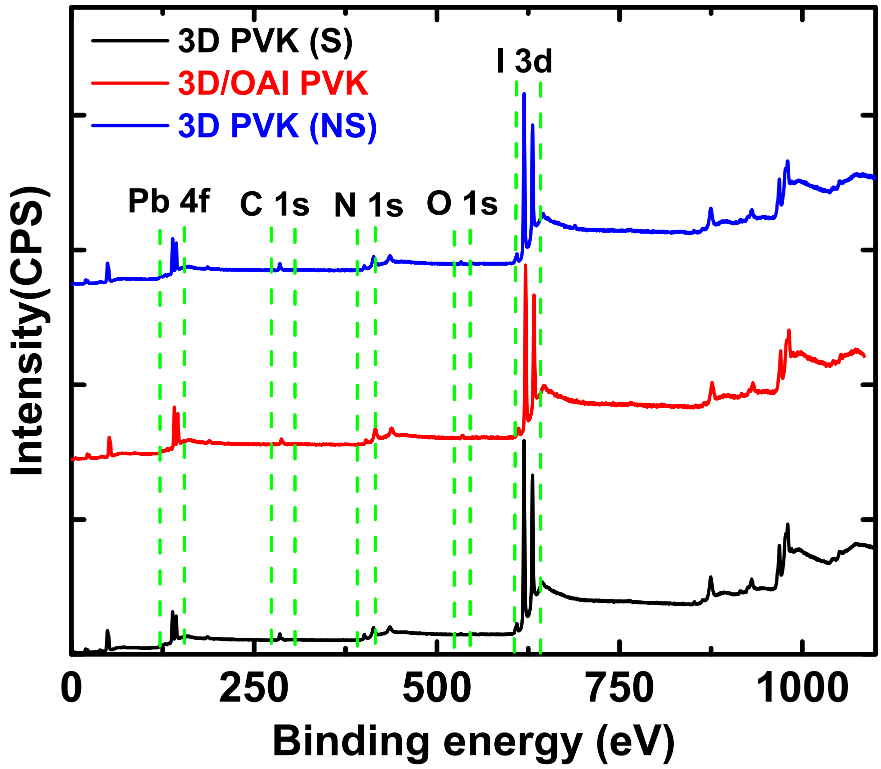

Figure S4. Survey spectra measured by XPS showing the surface composition of the perovskite film PVK (S) without OAI (black) and with OAI (blue) and PVK (NS) perovskite film without OAI (red).

Table S2. Table showing the percentage of elements from the surface of the different perovskites measured by the XPS survey spectra.

Sample	Pb (%)	I (%)	C (%)	N (%)	O(%)
**3D PVK (S)**	8.93	40.43	37.1	12.4	1.1
**3D/2D PVK (S)**	9.3	41	38	7.6	0.1
**3D PVK (NS)**	10.4	36.9	39	9.3	4.1

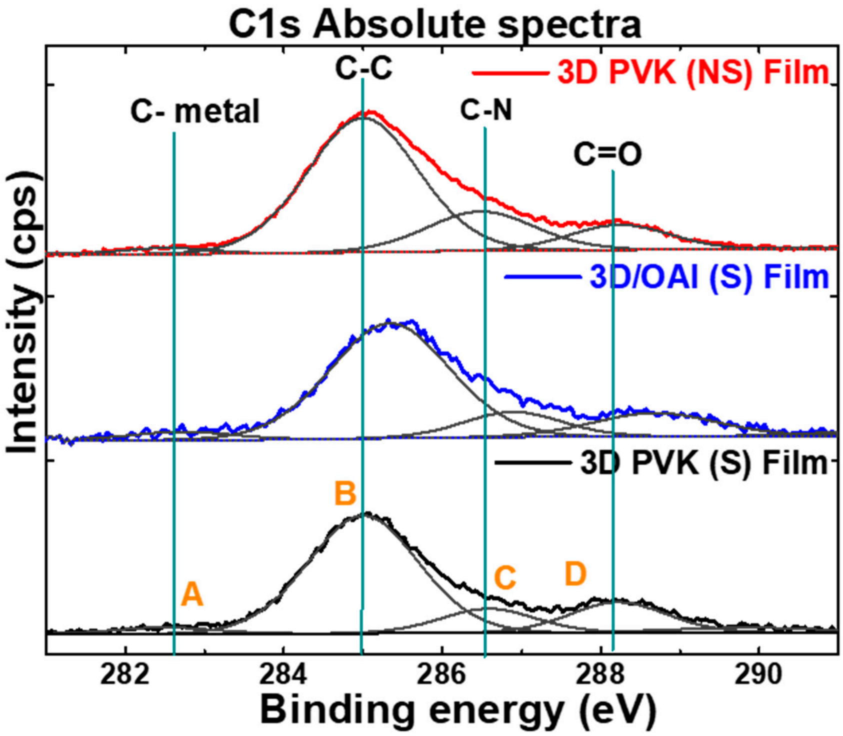

Figure S5. Absolute and deconvoluted spectra of core level of C (1s) measured by XPS for the perovskite film PVK (S) without OAI (black), perovskite film PVK (S) with OAI (blue), and perovskite film PVK (NS) without OAI (red) showing the different bonds formed by C.

.

Table S3. Table showing the proportion of the different bonds formed by C with other elements from the absolute spectra. The numbers were calculated after subtracting the background and standard peak fitting.

Sample	C-metal (%),A	C-C (%), B	C-N (%), C	C=O (%),D
**3D PVK (S)**	3.3	66.1	11.7	15
**3D/2D PVK (S)**	4.1	67	12	14.2
**3D PVK (NS)**	2.2	65.9	18.8	11.8

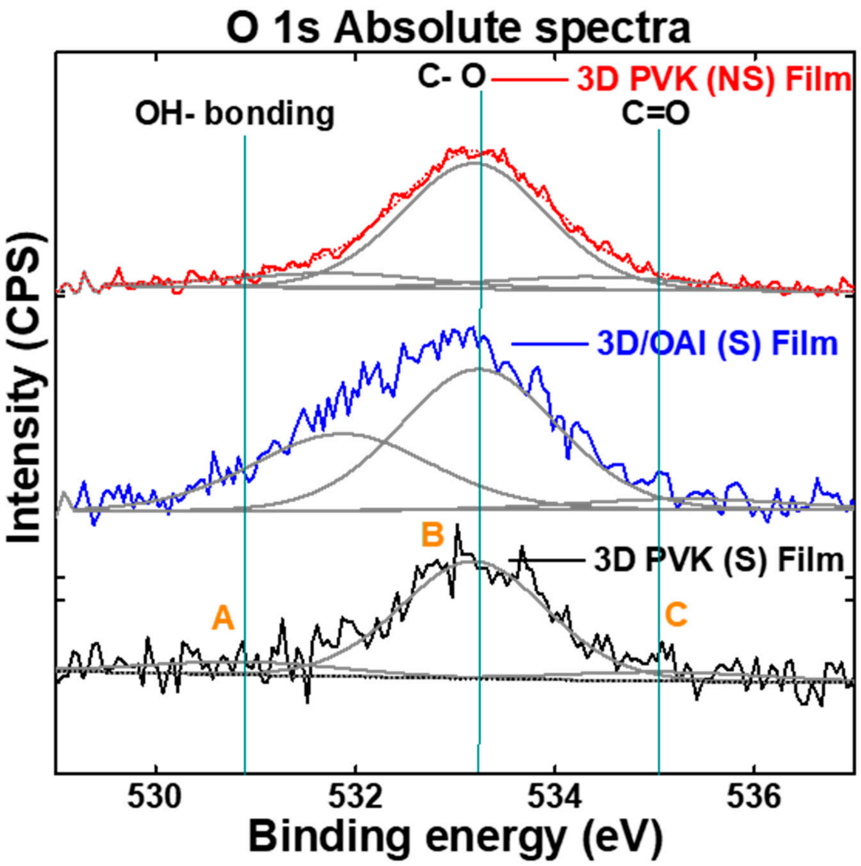

Figure S6. Absolute and deconvoluted spectra of core level of O (1s) measured by XPS for the perovskite film PVK (S) without OAI (black), perovskite film PVK (S) with OAI (blue), and perovskite film PVK (NS) without OAI (red) showing the different bonds formed by O.

Table S4. Table showing the proportion of the different bonds formed by O with other elements from the absolute spectra. The numbers were calculated after the subtracting the background and standard peak fitting.

Sample	O-H (%),A	C-O (%), B	C=O (%),C
**3D PVK (S)**	12.1	79.3	8.6
**3D/2D PVK (S)**	36.1	58.2	5.8
**3D PVK (NS)**	12.4	76	11.6

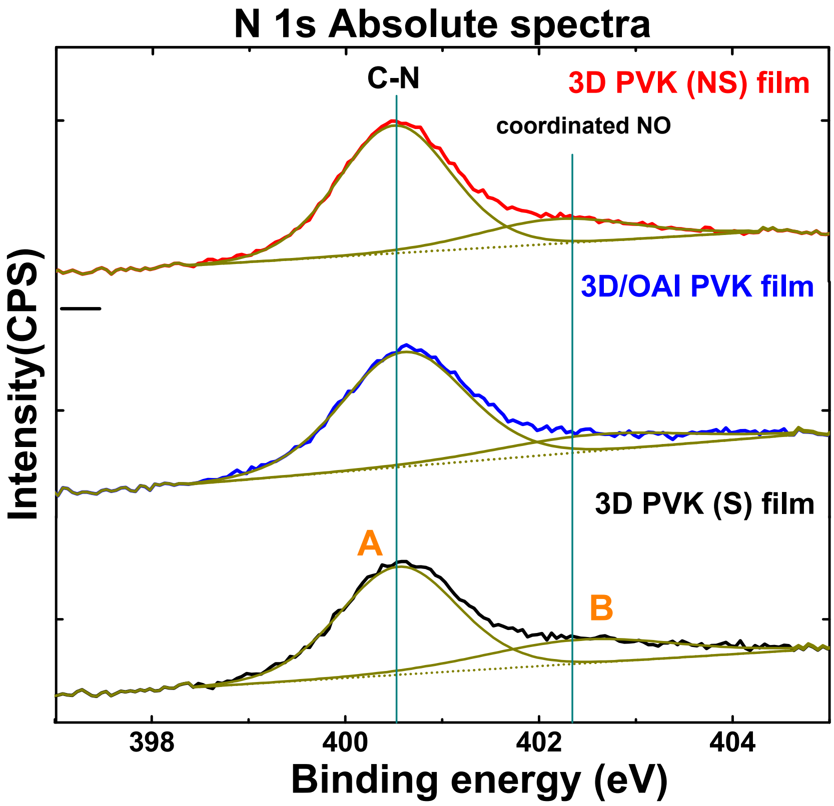

Figure S7. Absolute and deconvoluted spectra of core level of N (1s) measured by XPS for the perovskite film PVK (S) without OAI (black), perovskite film PVK (S) with OAI (blue), and perovskite film PVK (NS) without OAI (red) showing the different bonds formed by O.

Table S5. Table showing the proportion of the different bonds formed by N with other elements from the absolute spectra. The numbers were calculated after subtracting the background and standard peak fitting.

Sample	C-N (%), A	Coordinated NO, B
**3D PVK (S)**	73.7	26.3
**3D/2D PVK (S)**	81.1	18.9
**3D PVK (NS)**	79	21

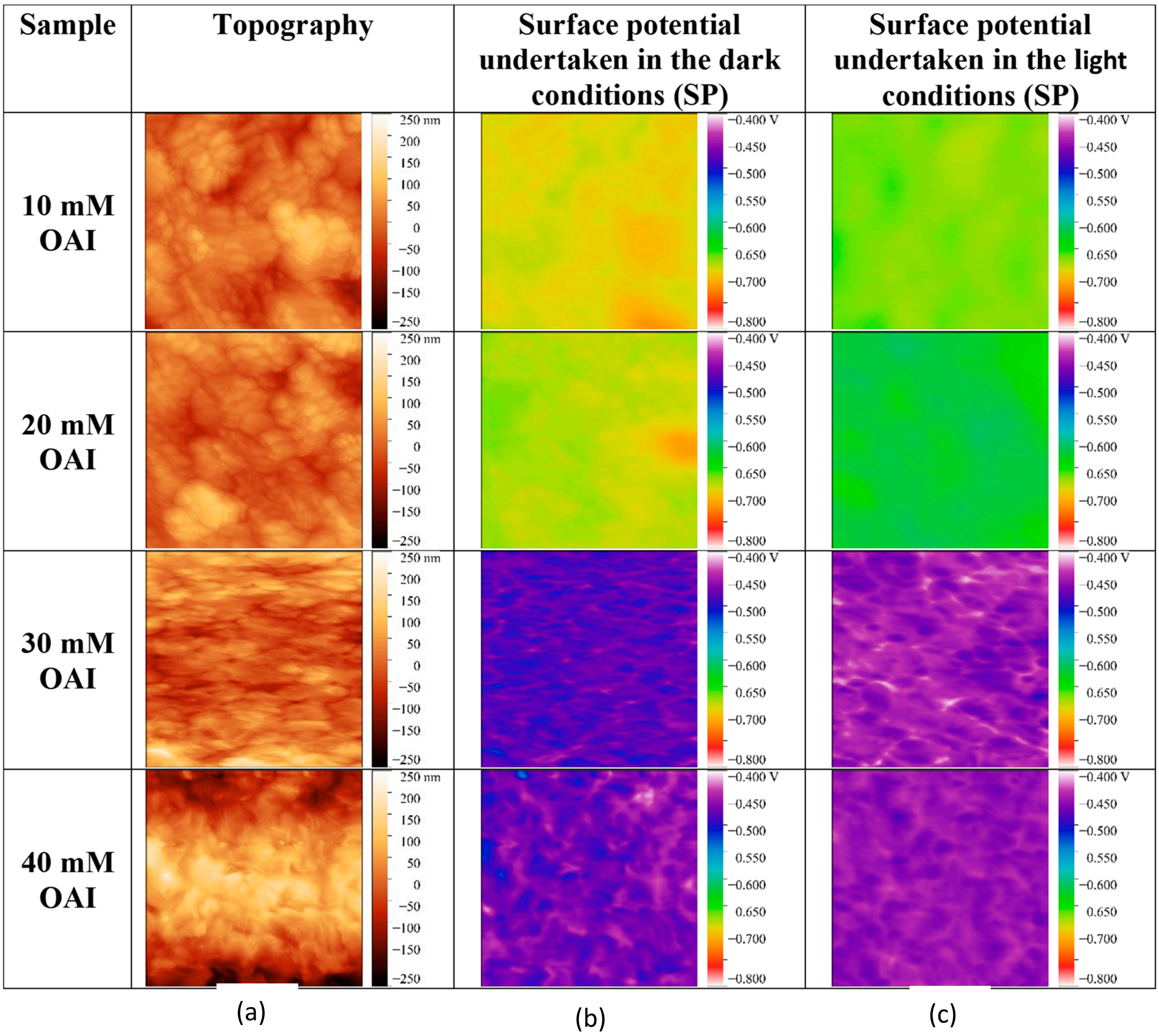

Figure S8. Topography of the perovskite films PVK (NS) prepared from a non-stoichiometric precursor containing different concentrations of OAI, measured by atomic force microscopy (a); color plots of the same films showing the distribution of surface potential measured from the surface (b,c). The measurements were taken in the dark and under light.

Table S6. Average surface roughness measured from AFM images and surface potential values of the perovskite films PVK (NS) with different concentrations of OAI measured in the dark and under white-light illumination.

Sample code	**Average roughness (nm)**	**Surface Potential under dark (V)**	**Surface Potential under Illumination (V)**
**No OAI or 0 mM**	29.0	-1.07	-0.984
**OAI (10 mM)**	38.8	-0.69	-0.667
**OAI (20 mM)**	37.7	-0.68	-0.623
**OAI (30 mM)**	49.0	-0.48	-0.451
**OAI (40 mM)**	85.4	-0.47	-0.456

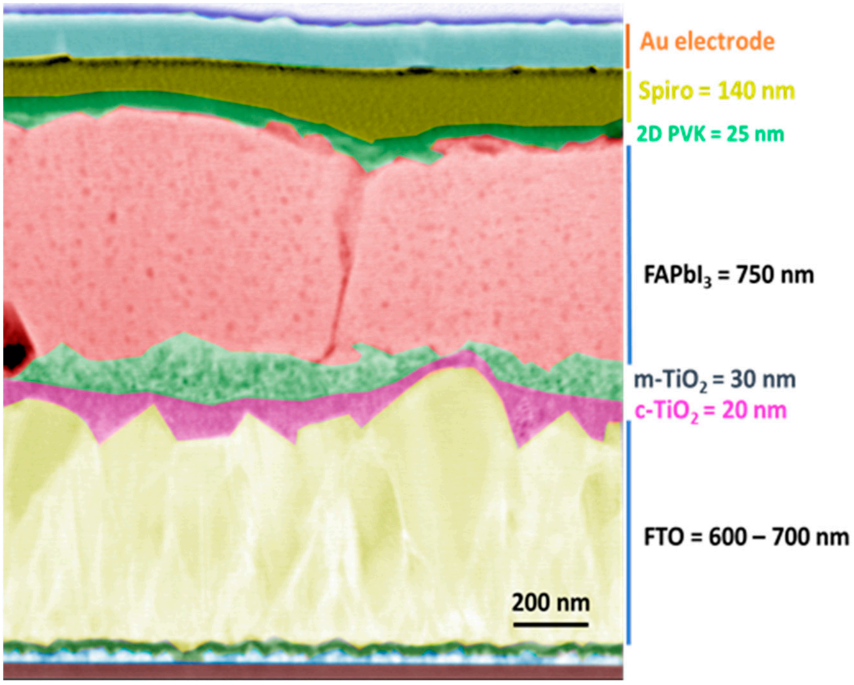

Figure S9. Color-marked cross-section TEM image of the champion solar cell made with an OAI concentration of 40 mM showing the thickness of all the layers in the stack.

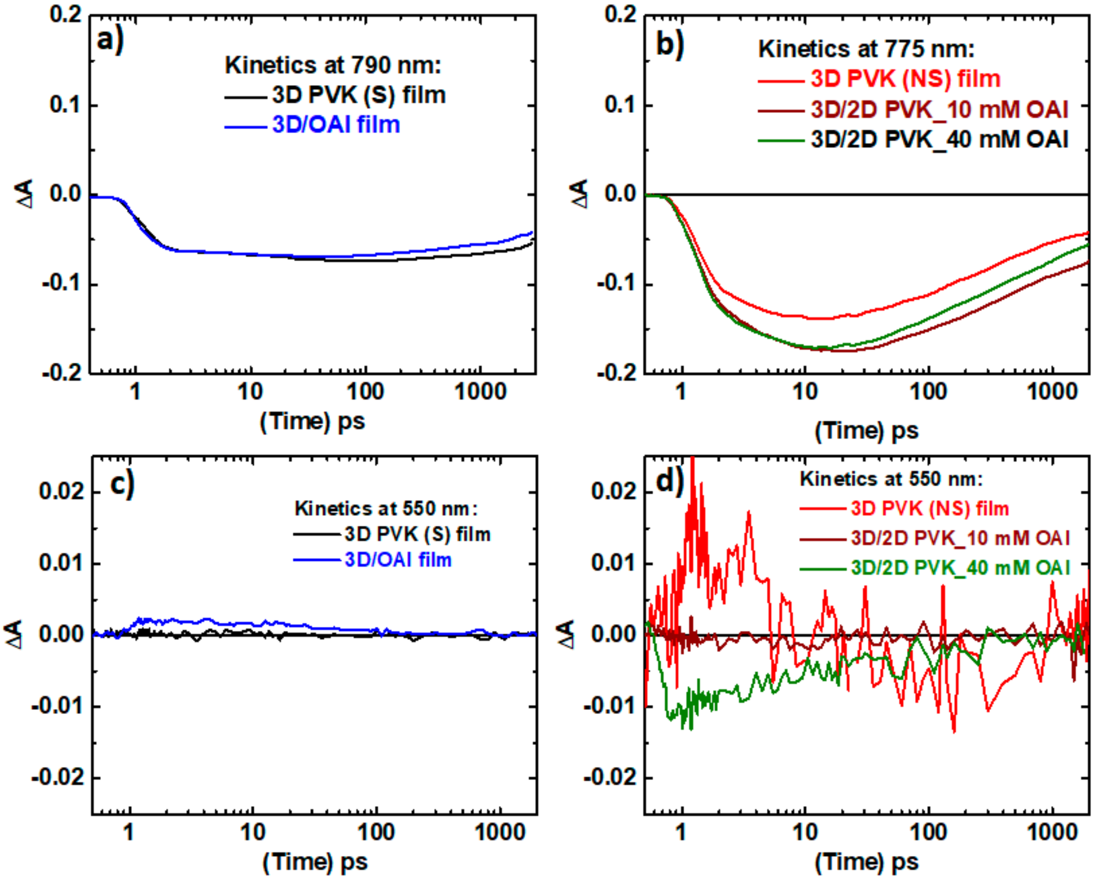

Figure S10. Kinetics of the transient absorption spectra measured for $FAPbI_3$ perovskite films from stoichiometric solution PVK (S), with and without OAI corresponding to bleaching at 790 nm (a); $FAPbI_3$ perovskite films from non-stoichiometric solution PVK (NS) with varying concentrations of OAI corresponding to the bleaching at 775 nm (b); $FAPbI_3$ perovskite films PVK (S), with and without OAI, corresponding to the bleaching at 550 nm (c); $FAPbI_3$ perovskite films PVK (NS) with varying concentrations of OAI corresponding to the bleaching at 550 nm (d).
